# Classification of short and long term mild traumatic brain injury using computerized eye tracking

**DOI:** 10.1038/s41598-024-63540-8

**Published:** 2024-06-03

**Authors:** Alice Cade, Philip R. K. Turnbull

**Affiliations:** 1 School of Optometry and Vision Science, The University of Auckland, Private Bag 92019, Auckland, 1023 New Zealand; 2 New Zealand College of Chiropractic, Auckland, New Zealand

**Keywords:** Reproducibility of results, Post-concussion syndrome, Ocular motility disorders, Brain concussion, Machine learning, Brain injuries, Diagnosis

## Abstract

Accurate, and objective diagnosis of brain injury remains challenging. This study evaluated useability and reliability of computerized eye-tracker assessments (CEAs) designed to assess oculomotor function, visual attention/processing, and selective attention in recent mild traumatic brain injury (mTBI), persistent post-concussion syndrome (PPCS), and controls. Tests included egocentric localisation, fixation-stability, smooth-pursuit, saccades, Stroop, and the vestibulo-ocular reflex (VOR). Thirty-five healthy adults performed the CEA battery twice to assess useability and test–retest reliability. In separate experiments, CEA data from 55 healthy, 20 mTBI, and 40 PPCS adults were used to train a machine learning model to categorize participants into control, mTBI, or PPCS classes. Intraclass correlation coefficients demonstrated moderate (ICC > .50) to excellent (ICC > .98) reliability (*p* < .05) and satisfactory CEA compliance. Machine learning modelling categorizing participants into groups of control, mTBI, and PPCS performed reasonably (balanced accuracy control: 0.83, mTBI: 0.66, and PPCS: 0.76, AUC-ROC: 0.82). Key outcomes were the VOR (gaze stability), fixation (vertical error), and pursuit (total error, vertical gain, and number of saccades). The CEA battery was reliable and able to differentiate healthy, mTBI, and PPCS patients reasonably well. While promising, the diagnostic model accuracy should be improved with a larger training dataset before use in clinical environments.

## Introduction

Traumatic brain injury (TBI) is the alteration of typical brain function that occurs after even minor trauma to the head^1^. There are a range of etiologies and variable presentations of mTBI, whose diagnosis is usually clinical, despite debate around the definition of mTBI^2–4^. Understandably, there is interest in developing biomarkers or automated objective assessments for assisting with diagnosis and tracking recovery^5,6^. While questionnaires^7^ and blood biomarkers^8^ show promise for TBI detection and tracking, biomarkers are not commercially available, and questionnaires are subjective and can lack sensitivity especially for milder presentations^8,9^. For example, the eye tracking-based King-Devick test has high sensitivity (86–98%) and specificity (90–96%) for moderate and severe TBIs^10,11^, but is much poorer at identifying the more common mild TBI (mTBI)^9^. This is problematic as many as 15% of people with mTBI have symptoms that last longer than the normal three-month recovery time, termed persistent post-concussion syndrome (PPCS)^12^. Those who progress to PPCS are thought to have several factors predisposing them to long-term symptoms such as anxiety, depression, lower socioeconomic status, reduced coping ability, pain, and motivation^12^. While some predisposing factors to PPCS implicate a psychological origin, the symptoms of PPCS are measurable and quantifiable, and include problems with response inhibition or executive control, visual attention, and visuospatial processing^13^. Those with PPCS can exhibit impaired oculomotor function when compared to those with a similar injury severity but who experience a more rapid recovery^14^. Being able to predict those who are at risk of PPCS would be hugely beneficial for health care systems, but first markers need to be identified.

Post-mTBI oculomotor, attentional, and exteroceptive defects can occur, but one of the most common complaints post-mTBI is that of visual disturbances, with prevalence rates between 30 and 90%^15,16^. Eye movements are neurologically complex actions, involving many areas of the brain, so unsurprisingly functional visual defects are a common finding following all severities of TBI^15,17^. Visual defects can result from damage to the primary visual pathway to the occipital lobe, along the parieto-occipital pathway, frontal lobes, cerebellum, or to the oculomotor cranial nerves themselves^18^. Furthermore, eye movements also require integrated action from the proprioceptive^19^, attentional^20^, visual salience^21^, and vestibular^22^ brain centers, so injury to any of these areas can also lead to visual abnormalities^21,23^. Eye tracking has been used to highlight many defects in those with mTBI, including poorer fixation^24^, increased gaze-error during pursuit movements^25^, increased saccade latency^26,27^, reduced vestibulo-ocular reflex (VOR) gain^28^, poorer performance on the Stroop test^29^, and higher egocentric localisation errors^30,31^, but no one test is diagnostic. Therefore, a comprehensive battery of computerized eye tracking assessments (CEAs) would seem to be ideally positioned to objectively detect mTBI after an injury^32^. Previous researchers have created eye tracking batteries that are sensitive and specific to identifying mTBI^33^, but they require restrained participants or skilled clinicians to operate and are either expensive or no longer unavailable^34–36^. While using CEAs to identify those with mTBI is not a novel concept, disentangling the differences between healthy people, mTBIs, and those with PPCS is more complex. This study aimed to assess the feasibility of developing a machine learning model that used the outputs from a battery of CEAs to firstly differentiate those with mTBI from controls, and then attempt to identify differences in CEA results between recent mTBIs (symptomatic, but under three months since injury) and those with PPCS (mTBI symptoms present for over three months). This project is the first step towards building normative and brain-injured data sets from which accurate diagnoses can be made, as well as close measurements of injury change over time.

## Methods

### Trial design and participants

Baseline data from 55 control, 20 mTBI, and 40 PPCS participants were pooled from baseline measures across three other studies that used the same CEA battery and test regime.The first assessedthe test–retest reliability of the CEA tests in healthy people, the second assessed the effects of a vibratory intervention on the cervical spine in controls and mTBI, and the third was a randomised controlled interventional trial for people with PPCS^37^. All data used for this study were pre-intervention (i.e., baseline) measures from participants. The control group had no history of TBI, self-reported normal vision, and no known oculomotor deficits. All participants with mTBI or PPCS were diagnosed by their primary health care provider prior to enrolment, had self-reported normal vision (other than symptoms that started after the brain injury), and were able to understand the study information and consent processes. Additionally, a subset of the healthy control group had the test battery repeated (with a shuffled test order) in the same session, to evaluate CEA test reliability.

Participants were free to withdraw from the studies at any stage and gave written informed consent. All experimental protocols and procedures were approved by the New Zealand Health and Disability Ethics Committee (HDEC 19/CEN/130) and the studies complied with the Declaration of Helsinki.

### Materials/computer set-up

A laptop mounted eye tracker (Tobii 5, Tobii Group, Stockholm, Sweden, see Supplementary Materials) was used to record binocular gaze (133 Hz) and head position data (33 Hz) samples per second. Visual stimuli were presented on a laptop computer (Surface Book 2, Microsoft, Redmond, USA), and the eye tracker was calibrated using the Tobii calibration software for each participant prior to running the CEAs. During testing, the participant sat approximately 70 cm from the screen, although the CEAs accounted for changes in angular extent due to changes in viewing distance. The target stimuli consisted of a black cross-shaped target presented on a white background^21^. The tracker used did have a lower sampling rate than what is generally considered the minimum (240 Hz) for saccades^38^. Lower sampling rates can lead to aliasing of peak saccade velocity, affecting results. Similarly, over filtering the data can lower peak velocity, especially in data recordings with lower sampling rates^39^, hence why a filter was not used on these data. While some research suggests that good approximations of peak velocity can be obtained when modelling data from sampling rates as low as 50 Hz^40^, the decision was made to use multiple measures to investigate saccades, such as correctly performing a test or decision-making latency, rather than to solely rely on peak saccade velocity as an outcome.

### Computerized eye tracking assessments

Six tests with previously established diagnostic value in differentiating mTBI^32,37^, were used in this study.

**Figure 1:**
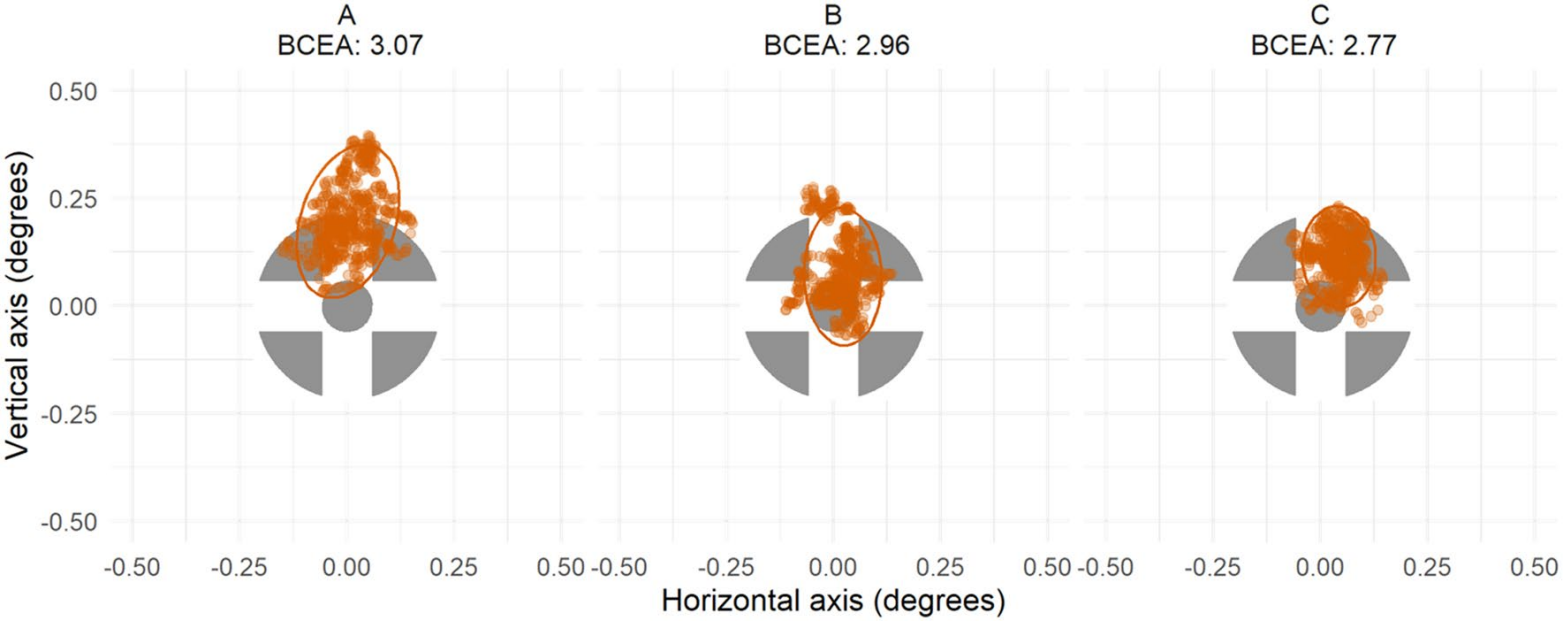
An example raw data from three trials of the fixation stability test. The orange points represent the participant’s gaze samples on the target (scaled size and opacity) when it was on the screen. The 95% bivariate contour ellipse area (BCEA, orange ellipse) is listed above each plot.

**Figure 2:**
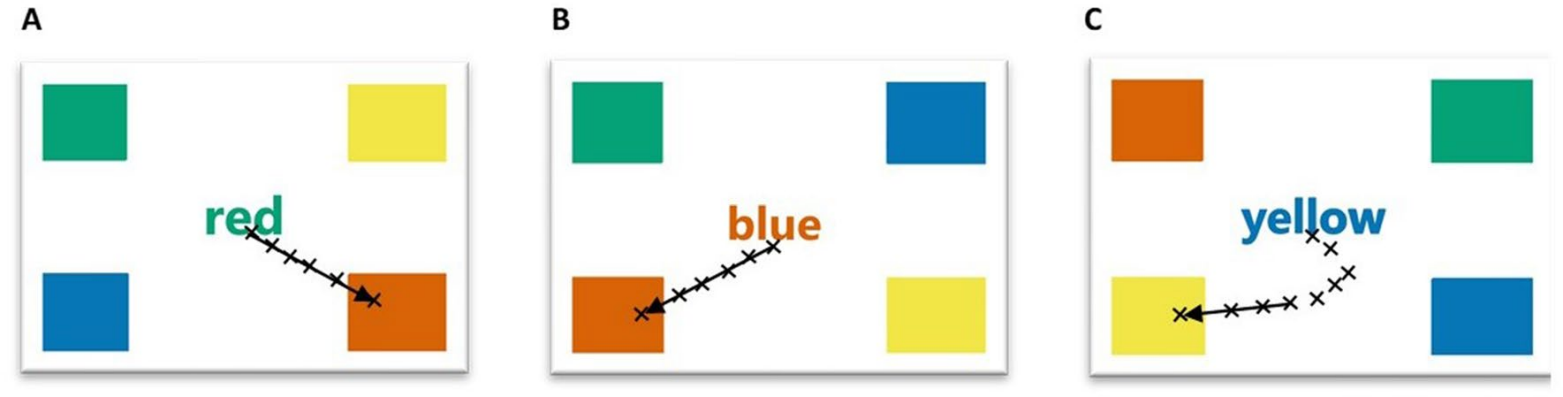
An example of the data from part one of the Stroop test, with X denoting gaze samples. Panel (**A**) shows gaze data from a single correctly performed part one trial, and panel (**B**) shows a correctly performed part two trial. Panel (**C**) shows a correctly performed part one trial is shown where the participant has corrected their gaze path partway through a trial.

1. *Egocentric localisation*. Participants were asked to move to align the center of their head with the target that appeared in a random screen location, ten times. The main outcomes were mean offset error and the mean trial completion time. The target was confined to the screen centre, with 15% screen width padding across the top, left, and right, and 30% padding from the bottom of the screen.
2. *Fixation stability*. Participants were instructed to maintain stable fixation on a centrally placed target for three trials of 10 s each. The main outcomes were the 95% bivariate contour ellipse area of fixation (BCEA, log_10_ minarc^2^)^41^, and a vector of mean gaze error, Fig. 1.
3. *Smooth pursuit*. Participants gaze followed a moving target as it traversed the screen in a Lissajous pattern, moving sinusoidally with a width of 11° and height of 6.5° at 10°/s for four trials of 30 s each. The outcomes measures were mean gaze offset error, total gain, and the number of catch-up saccades.
4. *Saccade test.* 14 pro-saccade and 14 anti-saccade tasks were randomly interleaved and assessed in one continuous run. In the saccade task the target appeared centrally on the screen for a random interval between 0.5–1.0 s. After this time the target color would change color to either green (for the pro-saccade task) or red (for the anti-saccade task) and stay visible for a further 0.5 s before disappearing. While the central target was still visible, a black secondary target, otherwise identical in size and shape to the main target, would appear at three degrees left or right of the screen for 0.2 s. Main outcomes were saccade latency—the time taken to decide where to move gaze after target presentation—and the number of correctly performed trials—did the participant look towards (pro-saccade task) or away (anti-saccade task) from the secondary target.
5. *The Stroop tests.* Adapted from the Stroop Color-Word test^29^. Part one involved directing gaze towards the color indicated by the stimulus word for 15 trials, then part two required participants to respond based on the stimulus font color, for another 15 trials (Fig. 2). Stroop outcomes included mean saccade decision making latency, total trial time, and proportion of correct trials. Prior to the test, participants viewed the 4 corner color blocks to familiarize themselves with the screen set-up. Each trial began with black words on a white screen of “Look here to start”, requiring active gaze for the trial to begin. For both parts a 50-point Arial font stimulus word appeared in the center of the screen. The stimulus words presented were “red,” “blue,” “green,” and “yellow,” randomly selected. The font color of each word was presented in red, blue, green, or yellow, however the word could not match the color of the font that the word was written in. For example, for the word red, the only possible font color permutations were green, blue, or yellow. The font colors were based on landmark colors that have unique psychophysiological bases, meaning they are easily recognized^42^. The colors used were adapted to color-blindness safe colors of blue (RGB: 0, 114, 178), red (RGB: 213, 94, 0), green (RGB: 0, 158, 115), and yellow (RGB: 240, 228, 66)^43^, and extended close enough to fixation that they did not need foveation to identify.
6. *The vestibulo-ocular reflex test*. The VOR was assessed with an active head impulse test^44^ by having the target appear centrally on the screen for 1.5 s, after this time the target turned green. The instructions given to each participant were to “Keep your eyes fixed on the central target as closely as possible then after it turns green, quickly turn your head to the left or right while keeping your eyes fixed on the central target.” There were 20 trials, 10 where participants turned their head to the left, and 10 to the right. Participants were free to choose which direction they first turned their head, then directed to alternate their head rotation in each subsequent trial^45^. Gaze was recorded for the first 15 degrees of head rotation. The main outcomes were fixation stability (as 95% BCEA), head to eye velocity gain, and the number saccades during each trial.

### Eye gaze data processing

Eye gaze data was captured continuously throughout the trials, and any trials where the participant moved out of the nominal measurement range of the eye tracker during the trial (e.g., closer that 500 mm or farther than 950 mm) had those trials excluded (only 4 trials were excluded throughout all data collection)^46,47^. Any trials with a saccade latency below 60 ms—5 trials total—were removed as the participants gaze was likely already moving, much like a false start in a running race, with an absolute minimum latency being previously reported at around 80 ms^48^. Gaze data, in screen coordinates, was converted to degrees of visual angle and gaze velocity was calculated as degrees of visual angle per second^49^. Blinks resulting in loss of signal were removed before analysis. As the Tobii 5 eye tracker has a sampling rate of 133 Hz, saccades were identified by time (at least 50 ms) and velocity (over 100 degrees/sec) to avoid misidentification of a saccade by velocity alone^50^.

### Statistical analyses

**Figure 3:**
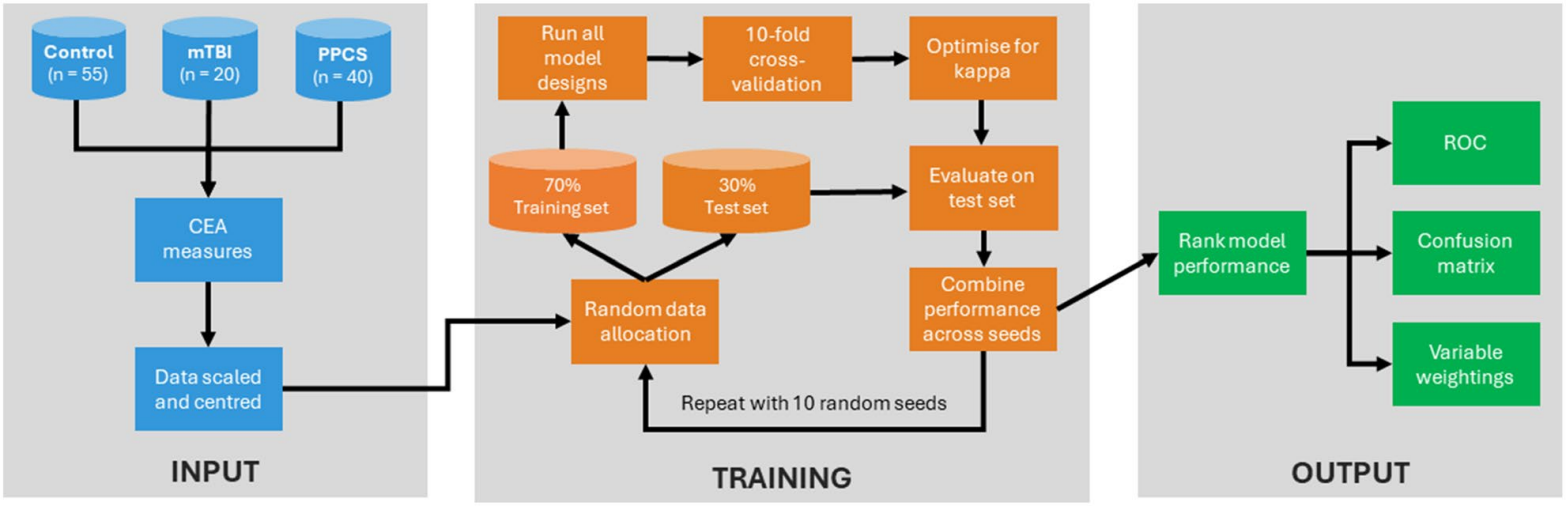
A schematic diagram showing the steps involved with training and evaluating the machine learning models.

Statistical analysis was performed using R software (version 4.0.3, https://www.r-project.org/) in RStudio (version 1.3.1093, https://posit.co/) using the stats, irr, and caret libraries. Bland–Altman plots with mean differences and limits of agreement^51^ and two-way intraclass correlation coefficients (ICCs) for consistency were used to assess reliability of CEA tests^52^. ICC values were described as excellent when greater than 0.90, good when between 0.75 and 0.90, moderate from 0.50 to 0.75, and poor when below 0.50^53–55^. For machine learning analysis, CEA outcome data were first normalized and centered, then with a common random seed, divided into a 70% training set and 30% test set. Each of the models were trained using 10 × cross-validation to prevent overfitting, using kappa, which compared to accuracy, accounts for the relative weightings of each class. The performance of each model and the relative importance of each variable was stored. To prevent chance overfitting to a specific random seed, the entire process was repeated using 10 different random seeds, which also enabled calculation of confidence intervals for model performance. To calculate ROC characteristics, pairwise ROC curves were computed with overall performance taken as a mean of all comparisons, Fig. 3.

Differences in CEA parameters between the three groups were compared using non-parametric Kruskal–Wallis test, statistical significance for all tests was set at p < 0.05 and adjusted using Holm multiplicity correction as appropriate^56^.

## Results

One hundred and fifteen age-balanced (control: 30.1 ± 7.9, mTBI: 25.5 ± 2.8, PPCS: 25.8 ± 4.3, χ^2^_(1, N=115)_ = 4.94,* p* = 0.085) and gender-balanced (female control: 65%, mTBI: 55%, PPCS: 70%, χ^2^_(1, N=115)_ = 3.53,* p* = 0.171) participants completed the whole experiment, with one mTBI participant withdrawing as the computer screen caused them painful photophobia. Their data was not included in the analysis.

### Repeatability of CEA measures

**Figure 4:**
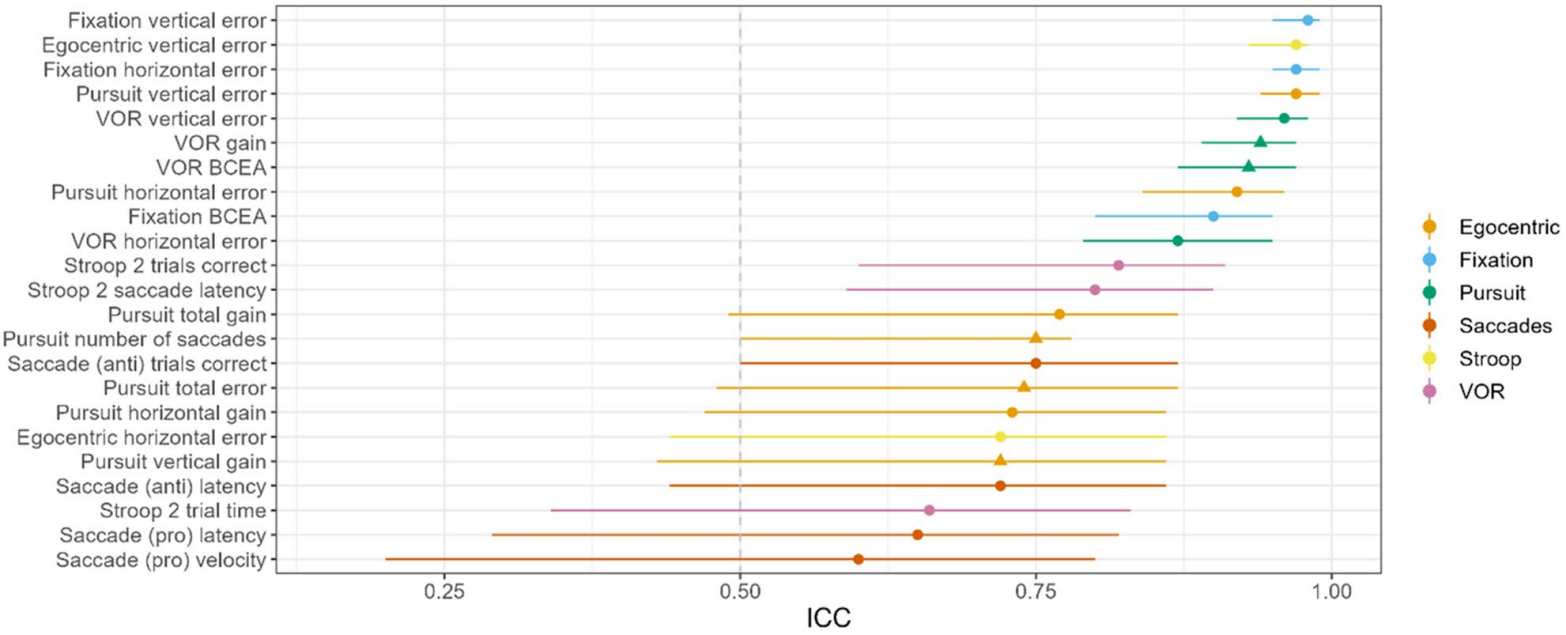
Intraclass correlation coefficients (ICC, points, with 95% confidence intervals) for all test outcomes with an ICC over 0.50. The outcomes that were ranked in the top five variables for the machine learning modelling have a triangle marker.

For ease of interpretation of the large number of test outcomes, all test results are provided in a Supplementary Table, and all outcomes with ICC values over 0.50 are shown in Fig. 4. Discussion of post-hoc comparisons is provided in the text below.

Analysis of gaze stability (BCEA and offset error) showed excellent reliability in fixation (ICC: 0.90–0.98) and VOR (ICC: 0.87–0.96). VOR gain reliability was also excellent (ICC: 0.94), but VOR time to maximum gain was poor (ICC: 0.24). Pursuit and egocentric localisation showed moderate to excellent reliability (ICC: 0.72–0.97).

The ICC for Stroop and saccade parameters was moderate for pro-saccade latency (0.65), velocity (0.60), anti-saccade latency (0.72) and anti-saccade correct trials (0.75), but poorer for pro-saccade correct trials (0.46) and anti-saccades velocity (0.46). Overall reliability in the Stroop test was higher for part two (ICC: 0.66–0.82), compared to part one (ICC: − 0.04–0.48). There was also some evidence of a learning effect during Stroop testing, with the probability of performing a trial correctly increasing with each trial for both part one (χ^2^(1, N = 35) = 0.72, *p* = 0.006) and two (χ^2^(1, N = 35) = 30.12, *p* < 0.001).

### Machine learning analyses

**Figure 5:**
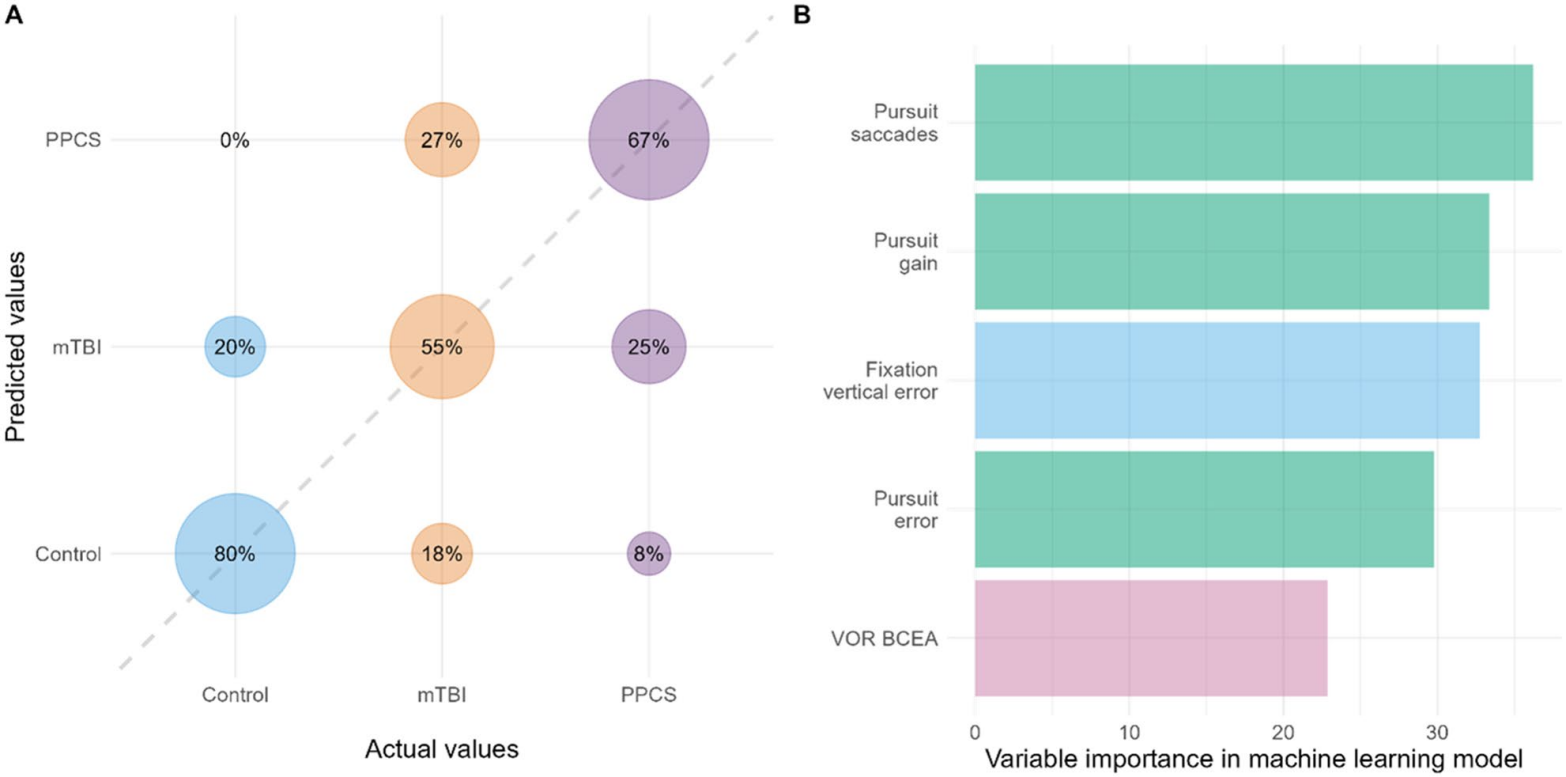
(**A**) Machine learning analysis confusion matrix for predicting control, mTBI, or PPCS. Overall, the balanced accuracy of 0.83, 0.66, and 0.76 for controls, mTBI, and PPCS, respectively. Further, the most common error in classifying mTBI was PPCS, and for PPCS the most common error was labelling as mTBI. As the distinction between mTBI and PPCS are arbitrarily defined as symptoms beyond three months, it is perhaps not surprising to see overlap between their symptomologies. (**B**) shows the top 5 outcomes that were most important to classifying groups in the machine learning model. There was no significant correlation between ICC values for outcomes and the importance of their weighting in machine learning modelling, Kendall’s correlation: τ_b_ = [0.25], p = .08.

Machine learning modelling was undertaken using a range of models to categorize participants into groups of control, mTBI, and PPCS. The most performant model used extreme gradient boosting with dropout (xgbDART), which yielded a mean kappa of 0.75 ± 0.20 and a mean 3-class pairwise AUC-ROC of 0.82 over the 10 random seeds. The balanced accuracy (mean of sensitivity and specificity) of the control, mTBI, and PPCS groups was 0.83, 0.66, and 0.76, respectively (Fig. 5A). This means that most brain-injured participants were correctly categorized as to their classification of injury (specificity mTBI: 0.77, PPCS: 0.88), and suggests the model was able to glean meaningful differences in the CEA data between groups. Key outcomes for modelling were the VOR (gaze stability), fixation (vertical error), and pursuit (total error, vertical gain, and number of saccades, Fig. 5B). While most CEA parameters had reasonable reliability, there was no correlation between each tests’ ICC value and the importance of their weighting in machine learning modelling (Kendall’s correlation: *τ*_*b*_ = 0.25, *p* = 0.08).

### Differences in CEA outcomes between groups

Gaze stability in VOR was different (H_(2)_ = 261.68, *p* < 0.001) between healthy and brain-injured participants (*p* < 0.001, control: 3.26 ± 0.94, mTBI: 3.94 ± 0.01, PPCS: 3.98 ± 0.90), but not between mTBI and PPCS (*p* = 0.930 mTBI: 3.94 ± 0.01, PPCS: 3.98 ± 0.90. Gaze error during pursuits was different between all groups (H_(2)_ = 73.16, *p* < 0.001) with mTBI (0.30 ± 0.23°) being significantly worse compared to controls (0.003 ± 0.26°, *p* < 0.001), whereas PPCS errors (PPCS: 0.15 ± 0.34°) were roughly midway between the mTBI and control means (*p* < 0.001). Vertical pursuit gain (H_(2)_ = 64.53, *p* < 0.001) was different between groups, with mTBIs having the highest gain (1.04 ± 0.03) compared to controls (1.03 ± 0.03, *p* = 0.008), while those with PPCS had a vertical pursuit gain closest to the ideal of 1.00 (1.02 ± 0.04). Also during pursuit testing, the number of catch-up saccades during a trial varied between groups (H_(2)_ = 21.35, *p* < 0.001), with the mean number saccades per trial being 0.50 ± 1.00, 0.38 ± 0.70, and 1.05 ± 1.61, for control, mTBI and PPCS, respectively. The number of saccades was not different between mTBI and controls (*p* = 0.470) but was between controls and mTBI (*p* = 0.001) and controls and PPCS (*p* < 0.001). For vertical offset error in fixation (H_(2)_ = 67.08, *p* < 0.001), only the PPCS group was significantly different (0.32 ± 0.75°), with their gaze sitting farther above the target compared to other groups (control: − 0.29 ± 0.47°, mTBI: − 0.38 ± 0.80°, *p* < 0.001). All significant and non-significant differences between groups for all outcomes are shown in the Supplementary Table.

## Discussion

### Key findings

This study found moderate to excellent test–retest reliability for a range of CEA measures, that also had good diagnostic value for differentiating control, mTBI and PPCS cohorts. Our results allowed a machine learning model can be trained with performance equivalent or better than previous eye tracking test batteries for mTBI^33–35^, even with a modest sample size. The CEA tests used in this study were specifically designed to be simple to use and to not require complex hardware or a highly trained clinician to administer.

### Most informative tests outcomes

Both the VOR and pursuit test had test parameters that were important in differentiating between the three classes, and the parameters that were most informative for the modelling tended to be different between classes using frequentist statistics. As the machine learning model also looks for complex relationships between variables, at least with our dataset it seems that such correlative measures or interactions did not add significant predictive value to the model. This may mean that the size of the model could be reduced with minimal impact on accuracy, allowing it to run on a wider range of hardware.

In terms of why VOR and pursuit may be most informative, it is important to note that in this study participants’ heads were not restrained. This permitted more naturalistic head movement and vestibular input during testing, and both VOR and pursuit require more complex cognitive integrations (including from VOR) before the resultant eye movement output. Proprioceptive drive from head rotation in the VOR test impacts gaze stability via cerebellar-vestibular pathways, whereas pursuit movements are mediated by predictive cerebellar-frontal pathways^57,58^. Perhaps, it is the combination of these two pathways—vestibular and frontal—for gaze stability and pursuit that uncover brain-injury deficits more effectively compared to simpler tests, such as saccades, which does not include as much proprioceptive input or prediction of movement. The VOR test’s importance may also further implicate the role of the injured cervical spine in mTBI symptomatology due to their similar mechanism of injury^59^. Cervical afferents, especially those of the upper cervical spine, heavily influence vestibulo-cerebellar components of gaze stabilization pathways and provide a secondary estimate of head movement, augmenting VOR function^60^. Disordered cervical proprioception post-injury may have affected brainstem activation or vestibulo-cerebellar components of the VOR pathway impacting its function^57,58,61^.

During pursuit movements, neurons in the vestibular nuclei receive direct cerebellar floccular input and provide premotor input to extraocular muscles helping to coordinate eye and head movement^62^. Due to the latency in the visual system, accurate pursuit requires anticipating target movement and learning target patterns, which originate from the middle temporal area and medial superior temporal area, and receive inputs from the frontal eye fields^63^. These areas, collectively, can be hypo-perfused and exhibit reduced glucose metabolism post-mTBI^64^, which is suggestive of poorer function and potentially explains the importance of pursuit gaze error in identifying mTBI. Poor gaze error would then lead to a need to catch the gaze up to the target with saccades, as was found in those with PPCS, further augmenting pursuit in mTBI classification and differentiating chronic symptomatology from recent injury.

During fixation testing, vertical error was of importance, which may also implicate cervical spine injury. Previous research showed that vibration to the cervical spine in mTBI worsened vertical fixation error, but a manual therapy aimed at reducing proprioceptive dysfunction in the spine after head injury improved vertical error^37^. These data suggest that just augmenting proprioceptive drive from the post-head-injured spine is not the key for improving fixation, but that reducing disordered proprioception is crucial.

Further information can also be gleaned from the errors that the model made, of which the most common was a misclassification between mTBI and PPCS. As the distinction between these two groups is an arbitrary period, the model may have identified participants that were heading towards chronicity of symptoms, and PPCS participants may have been returning to normality. It is possible that some outcomes tested may also help differentiating mTBI from PPCS specifically, such as the gaze error during pursuit testing, which was poorer in mTBI and better in PPCS, or the number of saccades in pursuits, which was significantly poorer in PPCS compared to the other groups. These data may guide further research into differentiating chronic from recent brain-injury, but more work is required as mechanism of injury or predisposing factors could also explain these differences.

### Performing trials correctly

Our pro-saccade latency (ICC = 0.59) and anti-saccades latency (0.72) ICC values were similar to previous findings (pro: = 0.66–0.79^65–67^, anti: = 0.64–0.73^65–68^). The number of trials performed incorrectly showed lower reliability for pro-saccades than anti-saccades, other research shows similar trends of pro-saccade ICCs of error rates being much lower (0.14^65^) compared to anti-saccade error rates (0.79–0.91^65,67^). Some of these differences may be explained by whether the pro- and anti-saccade tasks were interleaved, which has been shown to increase error rates in healthy controls compared to blocked-tasks error rates^69^. This difficulty in switching between tasks may also have explained the markedly longer saccade latencies and smaller difference between pro- and anti-saccade latency seen in this study, compared to previous research^26^. Switching between dual-tasks inhibits excitatory drive from the frontal eye fields and inhibit reflexive pro-saccades—essentially making an anti-saccade easier to perform and reducing the difference between pro- and anti-saccade latency^70^. For the Stroop test, part two error was consistent with previous research^71,72^, but our Part one error rates were lower (~ 15%) than normative data (~ 30%)^71,72^, but with poorer reliability (ICC = − 0.04) than reported by others^73,74^. Further investigation shows this to be confusion between the green and yellow colors—potentially explaining why there appeared to be a learning effect during Stroop testing—which was corrected through a more comprehensive introduction^37^.

## Future work

Regarding future use of this battery of CEAs, while this study focused on whether it captured differences in eye movement behaviours between mTBI and control groups, an alternate method could be to assess for within-subject differences in performance from a pre-season baseline. Used in this way, this CEA battery could track an athlete over the course of many games to assess for markers of cognitive dysfunction, which may not reach the level of even a mild TBI. Further work to understand normal longitudinal variations in CEA performance would be required, but this could greatly increase the sensitivity of the tests for detecting cognitive impairments.

## Strengths and limitation of the study

While this study piloted a new CEA test battery, a larger population will allow for more robust conclusions about which outcomes can best differentiate and predict mTBI and PPCS will make the eye tracking battery a useful tool in clinical practice. Longitudinal data collection could also be useful, to investigate which predict recovery, persist the longest, and which are the most sensitive to intervention. Longitudinally following a participant from their initial injury throughout their recovery, while tracking CEA outcomes, would create a more detailed dataset and potentially allow a more accurate model to be built. It could also help to build a metric of injury magnitude by correlating CEA outcomes with injury type, injury mechanism, symptoms experienced, and recovery time. This future modelling could help to predict an individual’s progress, track their recovery, and provide objective data to inform treatment pathways.

A new metric of injury magnitude may also be required, and perhaps a method to classify their mechanism of injury. The Rivermead Post-Concussion Questionnaire^75^ was slated to be this metric but inclusions as a covariate made no difference to any statistical modelling. Historically, differentiating mild from moderate and severe mTBI is problematic^76,77^, as well as being able to account for injury magnitude may help to better predict which group a participant falls into.

The eye tracker itself was also a possible limiting factor, for example, peak velocity of saccades may have been affected by the sampling rate of the eye tracker as they were at the lower end of what was expected^38^. Previous research has suggested sampling rates below 240 Hz can lead to aliasing—and concomitant lower peak velocities^39^. It is possible that future studies, using a higher sampling rate when recording data, may find some differences in saccade peak velocities compared to this study—a possibility that could be explored in further research. Aliasing may also explain why normative data for VOR gain is lower (~ 0.95–0.98^78^) than what this study found. A likely explanation could be the lower sampling rate (133 Hz) of the eye tracker compared to the 500 Hz^79^ or 1000 Hz^44^ sampling rates used in previous work. Potentially, the lower sampling rate led to blunting of velocity peaks altering the overall gain calculation. Additionally, the sampling rate of head tracking with the Tobii eye tracker is 33 Hz^46^ which, as per gaze tracking, could have led to underestimating head peak velocity, further affecting gain calculations. To fully explore this possibility further study would be necessary comparing the eye tracker used in this study to one with a higher sampling rate on the same population, although this problem is likely to reduce over time with the implementation of more advanced hardware.

## Conclusions

The results from this study suggest that the battery of CEAs is reliable and can be used to reasonably accurately predict if a participant is a healthy control, has an mTBI, or has PPCS. These findings should be tempered by the fact that, while reasonable performance was obtained from our data, the diagnostic model was trained on a limited range and scope of information and larger training data sets are required before they can inform clinical practice.

### Supplementary Information

Supplementary Information.

## Data Availability

The data that support the findings of this study are available on reasonable request from the corresponding author.
